# Two-Dimensional Core-Shell Structure of Cobalt-Doped@MnO_2_ Nanosheets Grown on Nickel Foam as a Binder-Free Battery-Type Electrode for Supercapacitor Application

**DOI:** 10.3390/nano12183187

**Published:** 2022-09-14

**Authors:** Md Moniruzzaman, Yedluri Anil Kumar, Mohan Reddy Pallavolu, Hammad Mueen Arbi, Salem Alzahmi, Ihab M. Obaidat

**Affiliations:** 1 Department of Chemical and Biological Engineering, Gachon University, 1342 Seongnam-daero, Seongnam-si 13120, Gyeonggi-do, Republic of Korea; 2 Department of Physics, United Arab Emirates University, Al Ain 15551, United Arab Emirates; 3 National Water and Energy Center, United Arab Emirates University, Al Ain 15551, United Arab Emirates; 4 School of Chemical Engineering, Yeungnam University, Gyeongsan 38541, Republic of Korea; 5 Department of Chemical & Petroleum Engineering, United Arab Emirates University, Al Ain 15551, United Arab Emirates

**Keywords:** cobalt-doped manganese oxides, electrode, supercapacitors, energy storage, hydrothermal method

## Abstract

Herein, we present an interfacial engineering strategy to construct an efficient hydrothermal approach by in situ growing cobalt-doped@MnO_2_ nanocomposite on highly conductive nickel foam (Ni foam) for supercapacitors (SCs). The remarkably high specific surface area of Co dopant provides a larger contacting area for MnO_2_. In the meantime, the excellent retentions of the hierarchical phase-based pore architecture of the cobalt-doped surface could beneficially condense the electron transportation pathways. In addition, the nickel foam (Ni foam) nanosheets provide charge-transport channels that lead to the outstanding improved electrochemical activities of cobalt-doped@MnO_2_. The unique cobalt-doped@MnO_2_ nanocomposite electrode facilitates stable electrochemical architecture, multi-active electrochemical sites, and rapid electro-transports channels; which act as a key factor in enhancing the specific capacitances, stability, and rate capacities. As a result, the cobalt-doped@MnO_2_ nanocomposite electrode delivered superior electrochemical activities with a specific capacitance of 337.8 F g^–1^ at 0.5 A g^–1^; this is greater than pristine MnO_2_ (277.9 F g^–1^). The results demonstrate a worthy approach for the designing of high-performance SCs by the grouping of the nanostructured dopant material and metal oxides.

## 1. Introduction

The intemperate exploitations of fossil fuels have led us to energy consumption limits and unsustainable environmental difficulties [1,2,3]. Supercapacitors (SCs) are a new type of greener energy storing devices among batteries and capacitors that have the benefits of higher efficiency, larger power density, environmental protections, longer cycles, etc. [4,5,6]. However, the low energy densities of SCs limit their large-scale configuration commercially. An efficient route to enhance this energy density is to make asymmetric SCs [7,8,9].

Electrodes are one of the crucial elements influencing the performances of SCs [10]. Different types of material samples have been developed so far for achieving better energy densities, such as doped materials [11,12,13], metal hydroxides/oxides [14,15], composite electrodes [16,17,18,19], and conductive polymers [20,21,22,23]. From this perspective, MnO_2_ becomes a favored source for making pseudocapacitor (PCs) electrodes due to their superior theoretical capacities (~1370 F g^−1^), cheaper prices, and eco-friendliness [24]. MnO_2_ would deliver excellent capacities and characteristic features in neutral electrolytes, which do not need stronger acids or alkalic-type environments; in turn, this is good for the environment [25]. However, the MnO_2_ conductivity is still poor; this issue can be solved by combining MnO_2_ with a dopant material, which enables larger specific surface areas and excellent conductivities [26,27].

Cobalt-doped electrodes have features such as excellent electrical conductivities, good chemical stabilities, superior surface interfaces, and a cheaper price. Thus, it could be contemplated as an absolute candidate for supporting MnO_2_ in forming a composite electrode sample [28]. The fabrication of cobalt-doped materials on nickel foam skeleton has attracted a lot of interest so far [4,29,30,31]. Nickel foam is safer, greener, and plentiful; thus, it became favorable for renewable energy developments. Cobalt-doped material on the nickel foam skeleton generally consists of superior surface area and porosities, which would efficaciously shorten the ion/electron transportation intervals. Nickel foam with cobalt-doped material handled with alkali or acids also has a plentiful functional surfacing group, which are favored for electrochemical activities [32,33,34,35]. Thus, the use of cobalt-doped material with nickel foam and MnO_2_ has been anticipated to enhance the performances of both MnO_2_ and cobalt-doped materials.

Herein, we developed a unique cobalt-doped MnO_2_ with the conductive skeleton of nickel foam via a hydrothermal technique. The composite of cobalt-doped@MnO_2_ delivers excellent energy storing performance. This would be ascribed to the excellent retention of the conductive way and uniformly loaded MnO_2_. Cobalt-doped@MnO_2_ facilitated the self-assembly of the composites with nickel foam; meanwhile, the metal oxides constructively enhanced the capacities by transmitting the composite with PCs. The energy storage performances of the cobalt-doped@MnO_2_ nanosheets were synergistically developed, providing multiple chemical states of Co-existences in the electrode. The results manifested the specific capacitance of the cobalt-doped@MnO_2_ nanosheets is 337.8 F g^−1^at 0.5 A g^−1^; this surpasses composites in recently reported literature.

## 2. Experimental Procedure

### 2.1. Synthesis of Cobalt-Doped@MnO_2_ Composite Nanosheets

Before synthesis, nickel foam (2 × 1 cm^2^) was carefully cleaned with a 6.0 M HCl solution in an ultrasound bath for 30 min to remove and eliminate the influence of the NiO layer from the surface; it was then rinsed with deionized water and absolute ethanol several times; and finally, dried in a vacuum oven at 50 °C. Cobalt nitrate hexahydrate (3 g) was added to 65 mL of MnCl_2_ solution with a concentration of 0.034 mol L^−1^. Then, the precursor solution was continuously treated with ultrasound for 20 min to permit the complete adsorption of Mn^2+^ on cobalt nitrate hexahydrate. In due course, the supernatant liquids were detached by centrifugation; and 65 mL of KMnO_4_ solutions (0.069 M) were added to the mixtures. After stirring at 115 °C for 3 h, the precursor mixtures were washed with DI water continuously; and finally, dried at 130 °C for 12 h to gain the cobalt-doped@MnO_2_ nanosheets composite.

For comparison, pure MnO_2_ electrodes were also fabricated by a similar reaction process without adding Cobalt nitrate hexahydrate into the MnCl_2_ solution.

### 2.2. Characterizations

The electrode morphology was investigated by scanning electron microscopy (FE-SEM, S-4800, Hitachi, Busan, Korea) and transmission electron microscopy (HRTEM, CJ111). The elemental compositions and chemical states of the spectroscopic procedures of the electrode were studied by X-ray photoelectron spectroscopy (XPS, VG Scientific—ESCALAB 250, Busan, Korea.). The electrode sample structure of the crystal was perceived by X-ray diffraction (XRD, D/Max-2400, Rigaku, Tokyo, Japan, Cu Kα) at an acceleration voltage of 40 kV using Cu Ka (λ = 0.154 nm) radiation.

### 2.3. Electrochemical Measurement

The electrochemical activities of the electrode composites (MnO_2_ and cobalt-doped@MnO_2_) were investigated by a three-electrode configuration operating an electrochemical workstation (SP-150 Biologic instrument, Busan, South Korea) in a 2 M KOH electrolyte. The Pt wire (2 cm × 2 cm) and Hg/HgO electrode were employed as the counter and reference electrodes, respectively. The galvanostatic charge/discharge (GCD), cyclic voltammetry (CV) tests quantifications, and electrochemical impedance spectroscopy (EIS) analysis were captured with a counter and reference electrode. The EIS result was processed by operating AC potentials of 10 mV amplitude (versus Hg/HgO) in the ranges of 200 mHz to 200 kHz frequencies at open-circuit potential (OCP). For a three-electrode system, the AC potential of 10 mV was applied versus RE. The mass loading of the working sample on each electrode is ~3.4 mg. The specific capacitance (*C*_s_, F g^−1^) was calculated from the charge–discharge curve by using the following equation [34]:

*C*_s_ = (*I* × Δ*t)*/(*m* × Δ*V)* (1)

where *C*_s_, *I*, Δ*t*, and *m* are the specific capacitance (F g^−1^), current (A), discharge time (s), and mass (g) of the active materials, respectively.

## 3. Results and Discussion

**Figure 1:**
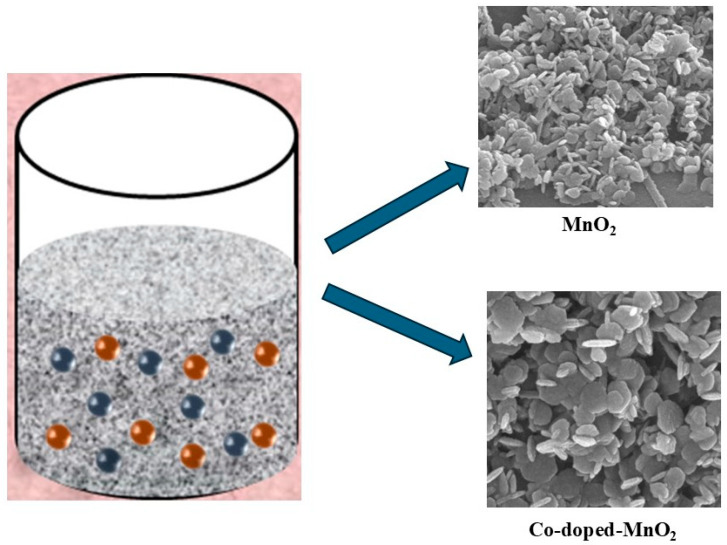
Schematic diagram of the cobalt-doped@MnO_2_ nanosheet composite.

Figure 1 is a brief illustration of the preparation procedures for the cobalt-doped MnO_2_ nanosheets composite. During the following hydrothermal procedures, the cobalt gradually transformed into a porous nickel foam structure. The cobalt dope supports the enhancement of the pore structures and provides the cobalt doping of the nickel form [29]. After a post in-situ hydrothermal deposition procedure, densely MnO_2_ nanoparticles were grown on the nickel foam. The chemical reactions involved in the procedure are as follows:

2MnO_4_^−^ + 3Mn^2+^ + 2H_2_O → 5MnO_2_ + 4H^+^ (2)

**Figure 2:**
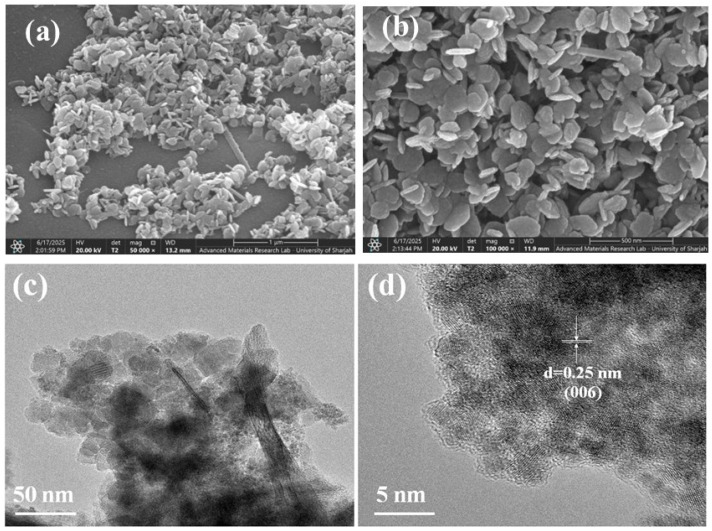
(**a**) SEM images of the MnO_2_ sample; (**b**) an SEM image of the cobalt-doped@MnO_2_ nanosheets composite; (**c**) a TEM image of the cobalt-doped@MnO_2_ nanosheets composite; and (**d**) an HRTEM image revealing the crystalline structure of the MnO_2_ nanosheets.

Figure 2a indicates a typical SEM image of the MnO_2_ nanoparticle material. The MnO_2_ nanoparticle appears with distinct porous architecture. The internally networked porous construction not only supplies a channel for quick electron transportations, but also acts as a well-being skeleton for the MnO_2_ loading. Afterward, the SEM images of the cobalt-doped@MnO_2_ nanosheets composite (Figure 2b) exhibits excellent dispersed MnO_2_ nanoparticles uniformly coating on the interfaces of the nickel foam. Figure S1 shows the SEM image of cobalt-doped@MnO_2_ nanosheets composite well-distributed on the nickel foam. It is visible that the porous nature of the nickel foam is well-retained; this not only encourages the electrolyte ion transportations, but also produces a superior contacting surface for the MnO_2_. The SEM structure analysis investigation obviously indicates that the cobalt-doped@MnO_2_ nanosheets structure was facilitated by the excellent electron transportations between the electrode surface area and electrolyte interface to enhance the electrochemical performance. The different crystalline faces with polycrystalline characterization exist; and there was an observable grain boundary among the MnO_2_ and cobalt dope in the structure of the cobalt-doped@MnO_2_. TEM images (Figure 1) display the surface of the cobalt-doped@MnO_2_ nanosheets composite, obviously disposing of dense nanoparticles loaded on the interfaces of the nickel foam. In addition, the HRTEM images (Figure 2d) of the MnO_2_ nanoparticles disclose spacing fringes of 0.25 nm; this correlates to the (006) MnO_2_ spacing planner. Further, the cobalt dope consists of numerous MnO_2_ that connect to form a highly porous network structure; which helps the electrolyte ions penetrate during the charge–discharge process.

**Figure 3:**
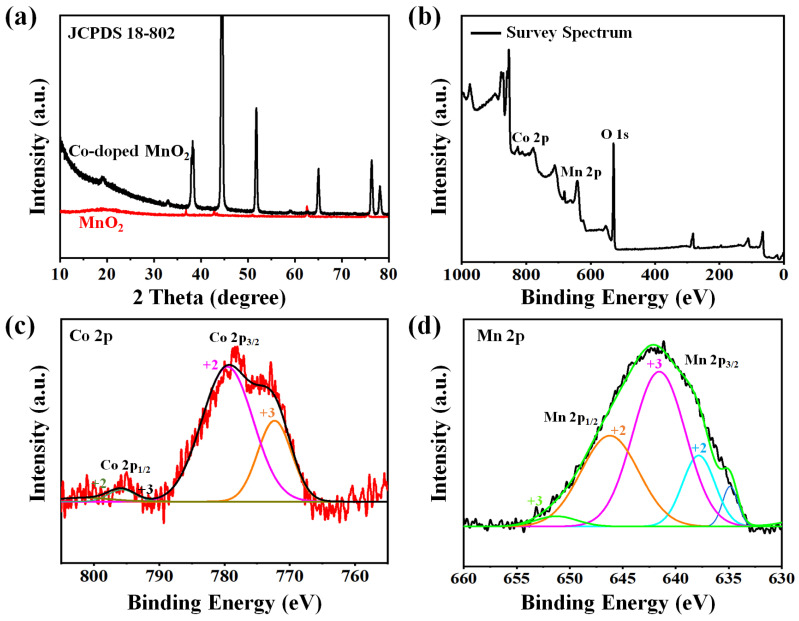
The XRD patterns (**a**) and wide-scan XPS spectra (**b**) of the cobalt-doped@MnO_2_ nanosheets composite, respectively. (**c**,**d**) The high-resolution XPS spectra for the cobalt-doped@MnO_2_ nanosheets composite of Co 2p and Mn 2p.

The crystalline structures of MnO_2_ and the cobalt-doped@MnO_2_ nanosheets composite were analyzed by XRD analysis, as depicted in Figure 3a. For the binary MnO_2_ material, two sharp peaks are visible around 22° and 43.5°; these are similar to that of the nickel foam [34,35]. For the cobalt-doped@MnO_2_ nanosheets composite, the three broader peaks at 12.3°, 36.8°, and 65.8° are correlated with (002), (006), and (119) planners of the birnessite category-MnO_2_ (JCPDS 18-802), respectively [36,37]. It is known that binary MnO_2_ is similar to MnO_2_ in the composite through comparisons.

XPS investigations were further employed to obtain information on elemental structure compositions, and the chemical molecular states of the surfaces of the electrode. Figure 3b is the general mapping of the XPS spectrum. The cobalt-doped@MnO_2_ nanosheets composite depicts the peaks of Co 2p, Mn 2p, C, and O elements. Furthermore, due to the loading of MnO_2_, the Mn peaks are visible in the spectra of the cobalt-doped@MnO_2_ nanosheets composite; and the C peaks decrease sharply. For the Co 2p XPS spectrum (Figure 3c), the spin-orbit split results of Co 2p_1/2_ (centered at 795 eV) and Co 2p_3/2_ (centered at 781 eV), transgression 15 eV; this reveals the coexistences of Co^3+^ and Co^2+^ cations [38,39]. By a Gaussian fitting method, the Co spectrum was fitted to four peaks, including the Co^3+^ peaks located at 780.8 eV and 781.1 eV and another peak located at 785.1eV and 802.5 eV, which was assigned to Co^2+^. For the Mn 2p pattern (Figure 3d), the two peaks at 643.5 eV and 655.6 eV correspond to Mn 2p_3/2_ and Mn 2p_1/2_, respectively [40,41,42]. The fitting peak at 637.7 eV is particularly characteristic of Mn^2+^, and the peaks located at 642.9 eV and 653.6 eV ascribed to Mn^3+^. The spin separations energies were 11.9 eV, which reveals that the Mn valence states were +4 [34,43,44]. The O 1s spectra would be deconvoluted into O-C (531.5 eV), C-O-C/C-OH (533.4 eV), and O–Mn (530.2 eV) bondings, respectively (as showed in Figure S2). The presence of the cobalt dope group was favored for the enhancement of electrochemical capabilities [32].

### Electrochemical Properties of Electrode Materials

**Figure 4:**
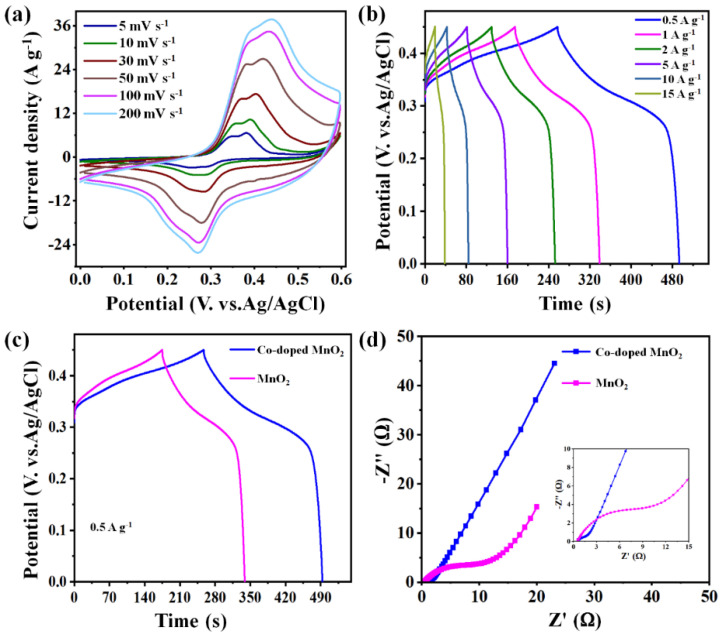
Electrochemical performances with the three-electrode system. (**a**) CV and (**b**) GCD curves of the cobalt-doped@MnO_2_ nanosheets composite. (**c**) GCD curves at a current density of 0.5 A g^−1^ of binary MnO_2_ nanoparticles and cobalt-doped@MnO_2_ nanosheets composite electrode materials. (**d**) The Nyquist plots of binary MnO_2_ and cobalt-doped@MnO_2_ nanosheets composite electrode materials.

The electrochemical capabilities of binary MnO_2_ nanoparticles and the cobalt-doped@MnO_2_ nanosheets composite were investigated using a three-electrode setup. The CV and GCD data values of the cobalt-doped@MnO_2_ nanosheets composite were depicted in Figure 4a,b, respectively. As the scan rates expand between 5 to 200 mV s^−1^, the CV plots remain in almost rectangular shapes; manifesting that the sample material consists of excellent reversibility and absolute capacitance nature. There were not any apparent redox peaks under the voltage windows of 0.0–0.6 V; which illustrates the behavior of the PCs of MnO_2_ and the PC nature of the cobalt-doped@MnO_2_ nanosheets composite. At various current densities between 0.5 A g^−1^ to 15 A g^−1^, the GCD pattern shows close symmetrical charge/discharges (Figure 4b). At a 0.5 A g^−1^ current density, we performed the comparison of both binary MnO_2_ nanoparticles and cobalt-doped@MnO_2_ nanosheets composite electrodes (Figure 4c). We also investigated the CV curve of binary MnO_2_ electrodes, as illustrated in Figure S3b. The CV curves comparison (Figure S3a) of the binary MnO_2_ nanoparticles and cobalt-doped@MnO_2_ nanosheets composite also display rectangular-type shapes. It is visible from the regions of the CV plots that the specific capacitances of the cobalt-doped@MnO_2_ nanosheets composite are greater than that of binary MnO_2_.

The data of EIS (Figure 4d) further evidenced that the cobalt-doped@MnO_2_ nanosheets composite consists of good electrochemical performances. The range in the frequencies of the pattern was from 0.02 Hz to 200 KHz. The Nyquist diagrams of binary MnO_2_ nanoparticles and cobalt-doped@MnO_2_ nanosheets composite electrode materials achieve the same small semicircles in the higher-frequency ranges (the semicircle diameter reveals charge transfer resistances (R_ct_)); manifesting that they have smaller charge transfer resistances. The R_ct_ of the cobalt-doped@MnO_2_ nanosheets composite was slightly greater than that of binary MnO_2_ nanoparticles. This was due to the charge transfer of cobalt-doped@MnO_2_ nanosheets presuming redox reactions, which were more moderate than the surface desorption/adsorption nature of the sample PCs [38]. In addition, it would be obvious that the cobalt-doped@MnO_2_ nanosheets composite effectively enhances the conductivities of binary MnO_2_ nanoparticles. The ideal capacitance character is apparent from the almost vertical linear plots in the lower frequency area. Thus, the cobalt-doped@MnO_2_ nanosheets sample reveals a much more oblique plot; this signifies the foremost performances of the PCs.

**Figure 5:**
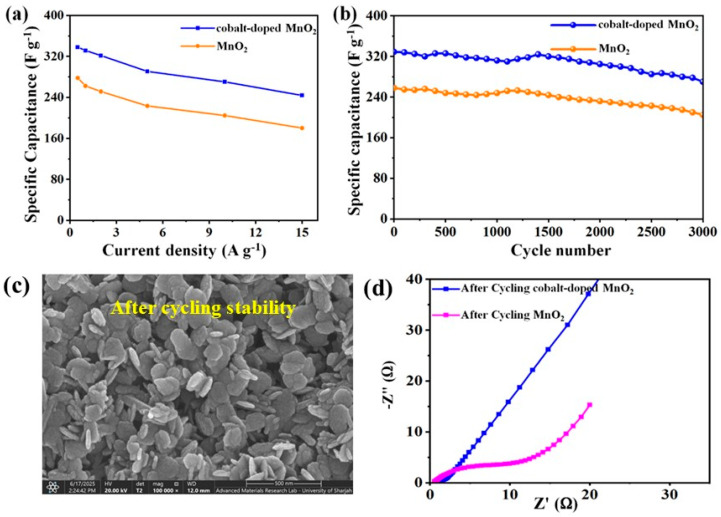
(**a**) Specific capacitances of binary MnO_2_ nanoparticles and cobalt-doped@MnO_2_ nanosheets composite electrodes; (**b**) charge–discharge cycling stability of binary MnO_2_ nanoparticles and cobalt-doped@MnO_2_ nanosheets composite electrodes at 2 A g^−1^; (**c**) an SEM image of the cobalt-doped@MnO_2_ nanosheets composite after 3000 long cycles; and (**d**) a Nyquist plot of after 3000 GCD cycles of binary MnO_2_ nanoparticles and cobalt-doped@MnO_2_ nanosheets composite electrodes.

Figure 5a displays the comparison of binary MnO_2_ nanoparticles and cobalt-doped@MnO_2_ nanosheets composite GCD plots at 0.5 A g^−1^, respectively. The specific capacitance of binary MnO_2_ nanoparticles was calculated to be 277.9 F g^−1^, which is approximately only 1/3 of the cobalt-doped@MnO_2_ nanosheets composite. These results illustrate that the porous behavior of the cobalt-doped@MnO_2_ nanosheets composite is advantageous to the electrolyte ions of diffusion. While conserving binary MnO_2_ nanoparticles’ PC capacitance, the cobalt-doped@MnO_2_ nanosheets composite electrode also expands PC capacitances.

According to the cycling test (Figure 5b), binary MnO_2_ nanoparticles consist of well- cycled stabilities; and the capacity retention rate residues ∼76.4% after 3000 long cycles. Whereas, for the cobalt-doped@MnO_2_ nanosheets composite, the electrode remains ∼82.5% after 3000 long cycles; manifesting that the nickel foam effectively enhances the cycling capabilities of MnO_2_. Surprisingly, both binary MnO_2_ nanoparticles and cobalt-doped@MnO_2_ nanosheets composite electrodes have excellent retention stabilities. The specific capacitance performances of the MnO_2_-based composite samples reported in previous studies are displayed in Table 1. The specific capacitances of the cobalt-doped@MnO_2_ nanosheets composite electrode are much higher than that of some nickel foam-based MnO_2_ composite materials and other MnO_2_-based composites. Figure 5c illustrates the SEM image of the cobalt-doped@MnO_2_ nanosheets composite material after 3000-long cycling stability. The SEM image shows a good surface structure and super-wettability, indicating a vital role in keeping faradaic redox and energy storage reactions. Figure 5d shows the impedance plots of the binary MnO_2_ nanoparticles and cobalt-doped@MnO_2_ nanosheets composite electrodes after 3000 cycles. There were no obvious changes of *R*_ct_ after 3000 long cycles, manifesting a rapid electron/ion transfer. Surprisingly, the higher performances of the cobalt-doped@MnO_2_ nanosheets composite with hierarchical structure on nickel foam is beneficial; owing to the larger surface area accessing point for ions that enhance the wettability of the composite and accelerate electron transfer.

## 4. Conclusions

In summary, cobalt-doped nanoparticles were uniformly grown on MnO_2_ with a large specific surface region and unique pore construction to form a cobalt-doped@MnO_2_ nanosheets composite. The improved electrochemical performances of the cobalt-doped@MnO_2_ nanosheets composite are ascribed to the higher electrical conductivities, enlarged surface region, ample working electrochemical sites, and rapid charging-transfer channels. The cobalt-doped@MnO_2_ nanosheets composite achieved extraordinary electrochemical capabilities. At a current density of 0.5 A g^−1^, the specific capacitance is 337.8 F g^−1^. Moreover, the cobalt-doped@MnO_2_ nanosheets composite electrodes exhibit excellent cycling stabilities of 82.5% capacity retention at 3000 GCD long cycles. The results of this research support the use of metal oxides as conductive bases and expand the scope of dopant-based material applications. Finally, the cobalt-doped@MnO_2_ nanosheets with the above unique physicochemical characteristics can have numerous good functionalities for other applications, such as biosensors, electrocatalysts, and batteries.

## Figures and Tables

**Table 1:** MnO_2_ composite electrode performance comparison over the last five years.

Electrode	Electrolyte	Specific Capacitance (F g^−1^)	Current Density (A g^−1^)	Ref.
MnO_2_/rice husk-derived composite	0.5 M Na_2_SO_4_	210.3	0.5	[30]
Holey reduced graphene oxide/MnO_2_ composites	1 M Na_2_SO_4_	192.2	0.5	[37]
MnO_2_@CCNs	1 M Na_2_SO_4_	262	0.2	[38]
CNT@NCT@MnO_2_	1 M Na_2_SO_4_	210	0.5	[40]
δ-MnO_2(4.0)_/HRGO	1 M Na_2_SO_4_	245	1	[45]
α-MnO_2_ NWs@δ-MnO_2_ NSs	6 M KOH	310.2	0.5	[46]
PPy/mesoporous MnO_2_	1 M Na_2_SO_4_	320	0.5	[47]
D-MNS-A@MnO_2_	1 M Na_2_SO_4_	231	1	[48]
cobalt-doped@MnO_2_ nanosheets	2 M KOH	337.8	0.5	This Work

## Data Availability

No new data were created or analyzed in this study. Data sharing is not applicable to this article.

## Supplementary Materials

The following supporting information can be downloaded at: https://www.mdpi.com/article/10.3390/nano12183187/s1, 1. Materials and Reagents; Figure S1. SEM image of cobalt-doped@MnO_2_ nanosheets well distributed on nickel foam; Figure S2. XPS spectra of O 1s; Figure S3a. CV comparison of binary MnO_2_ nanoparticles and cobalt-doped@MnO_2_ nanosheets composite; Figure S3b. CV curves of binary MnO_2_ nanoparticles. All authors have read and agreed to the published version of the manuscript.
