# Correction: Moniruzzaman et al. Two-Dimensional Core-Shell Structure of Cobalt-Doped@MnO_2_ Nanosheets Grown on Nickel Foam as a Binder-Free Battery-Type Electrode for Supercapacitor Application. *Nanomaterials* 2022, *12*, 3187

**DOI:** 10.3390/nano15231784

**Published:** 2025-11-27

**Authors:** Md Moniruzzaman, Yedluri Anil Kumar, Mohan Reddy Pallavolu, Hammad Mueen Arbi, Salem Alzahmi, Ihab M. Obaidat

**Affiliations:** 1 Department of Chemical and Biological Engineering, Gachon University, 1342 Seongnam-daero, Seongnam-si 13120, Gyeonggi-do, Republic of Korea; mani57chem@gachon.ac.kr; 2 Department of Physics, United Arab Emirates University, Al Ain 15551, United Arab Emirates; yedluri.anil@gmail.com (Y.A.K.); 201990023@uaeu.ac.ae (H.M.A.); 3 National Water and Energy Center, United Arab Emirates University, Al Ain 15551, United Arab Emirates; 4 School of Chemical Engineering, Yeungnam University, Gyeongsan 38541, Republic of Korea; pallavolumohanreddy@gmail.com; 5 Department of Chemical & Petroleum Engineering, United Arab Emirates University, Al Ain 15551, United Arab Emirates

In the published paper [1], there was an error in Figures 2 and 5c. The new schematic diagram (Figure 1), which is based on the new Figure 2, also requires correction. No changes were made to the conclusion or findings of the paper. The corrected Figure 1, Figure 2 and Figure 5 are shown below. This correction was approved by the Academic Editor. The original publication has also been updated.

## Figures and Tables

**Figure 1:**
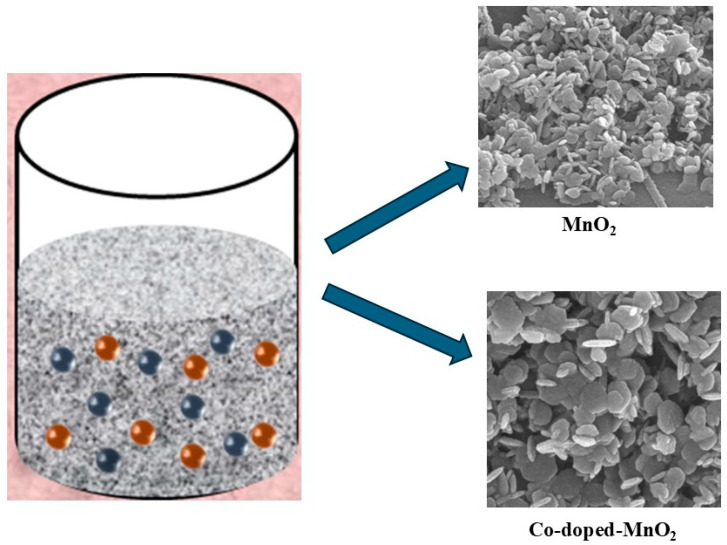
Schematic diagram of the cobalt-doped@MnO_2_ nanosheet composite.

**Figure 2:**
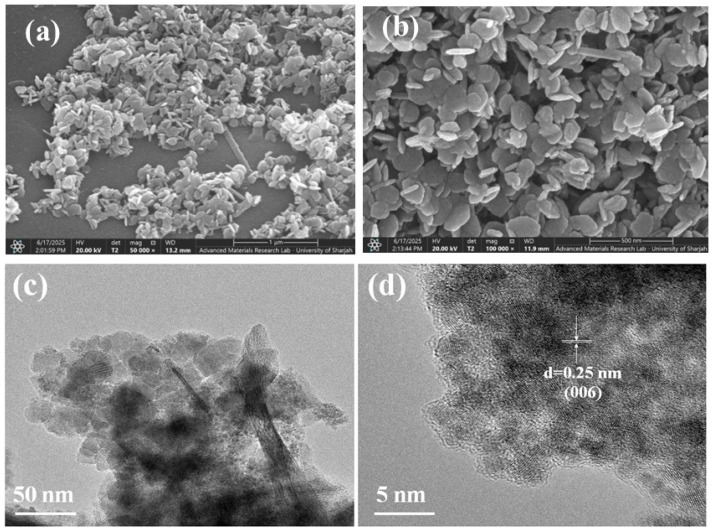
(**a**) SEM images of the MnO_2_ sample; (**b**) an SEM image of the cobalt-doped@MnO_2_ nanosheets composite; (**c**) a TEM image of the cobalt-doped@MnO_2_ nanosheets composite; and (**d**) an HRTEM image revealing the crystalline structure of the MnO_2_ nanosheets.

**Figure 5:**
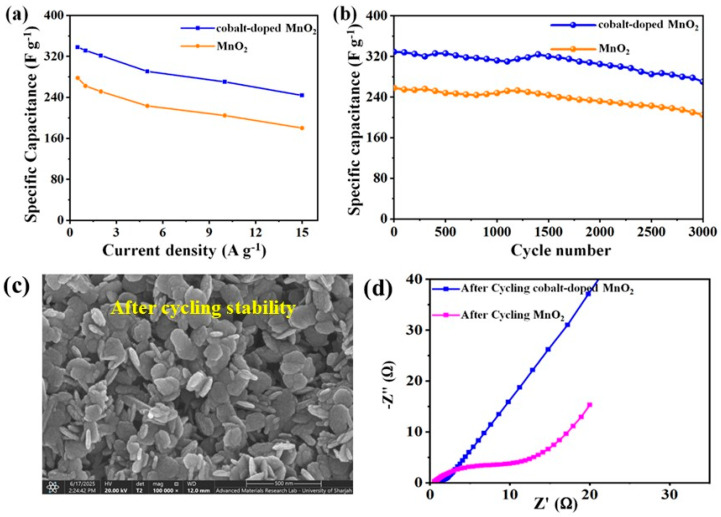
(**a**) Specific capacitances of binary MnO_2_ nanoparticles and cobalt-doped@MnO_2_ nanosheets composite electrodes; (**b**) charge–discharge cycling stability of binary MnO_2_ nanoparticles and cobalt-doped@MnO_2_ nanosheets composite electrodes at 2 A g^−1^; (**c**) an SEM image of the cobalt-doped@MnO_2_ nanosheets composite after 3000 long cycles; and (**d**) a Nyquist plot of after 3000 GCD cycles of binary MnO_2_ nanoparticles and cobalt-doped@MnO_2_ nanosheets composite electrodes.
