# Supplementary material for: Two-Dimensional Core-Shell Structure of Cobalt-Doped@MnO2 Nanosheets Grown on Nickel Foam as a Binder-Free Battery-Type Electrode for Supercapacitor Application
Source: Nanomaterials (Basel). 2022 Sep 14;12(18):3187. doi: 10.3390/nano12183187 (PMC9505914; doi:10.3390/nano12183187)
Supplement: Supplementary file 1 [file nanomaterials-12-03187-s001.zip › nanomaterials-1907641-supplementary.pdf]

*nanomaterials*

*Supplementary Materials*

# Two-Dimensional Core-Shell Structure of Cobalt-Doped@MnO$_2$ Nanosheets Grown on Nickel Foam as a Binder-Free Battery-Type Electrode for Supercapacitor Application

**Md Moniruzzaman [1], Yedluri Anil Kumar [2,3], Mohan Reddy Pallavolu [4], Hammad Mueen Arbi [2,3], Salem Alzahmi [3,5,*], Ihab M. Obaidat [2,3,*]**

[1] Department of Chemical and Biological Engineering, Gachon University, Gyeonggi-do 13120, Korea; mani57chem@gachon.ac.kr
[2] Department of Physics, United Arab Emirates University, Al Ain 15551, United Arab Emirates; yedluri.anil@gmail.com (Y.A.K.); 201990023@uaeu.ac.ae (H.M.A.)
[3] National Water and Energy Center, United Arab Emirates University, Al Ain 15551, United Arab Emirates
[4] School of Chemical Engineering, Yeungnam University, Gyeongsan 38541, Korea; pallavolumohanreddy@gmail.com
[5] Department of Chemical & Petroleum Engineering, United Arab Emirates University, Al Ain 15551, United Arab Emirates
* Correspondence: s.alzahmi@uaeu.ac.ae (S.A.); iobaidat@uaeu.ac.ae (I.M.O.)

## 1. Materials and reagents

All the chemicals of analytical grade were used as received without further purification. Manganese chloride hexahydrate ($MnCl_2 \cdot 4H_2O$, 99.1%, Sigma Aldrich, St. Louis, MO, USA), Cobalt nitrate hexahydrates ($Co(NO_3)_2 \cdot 6H_2O$, 99.2%, Sigma Aldrich), and urea ($CH_4N_2O$, 99.1%, Sigma Aldrich) were purchased from Sigma Aldrich, Korea. Ni foam has received from Japan. potassium hydroxide (KOH, 99.99%), Hydrochloric acid (HCl, 99.2%), and sodium hydroxide (NaOH, 99.8%) were purchased from Samchun Chemicals, Korea. Ni foam with a 1.6 mm thickness was purchased from Alantum, Korea. Deionized (DI, 100%) water and double-distilled water (DDW, 100%) were used especially for the purposes of preparing solutions and washing.

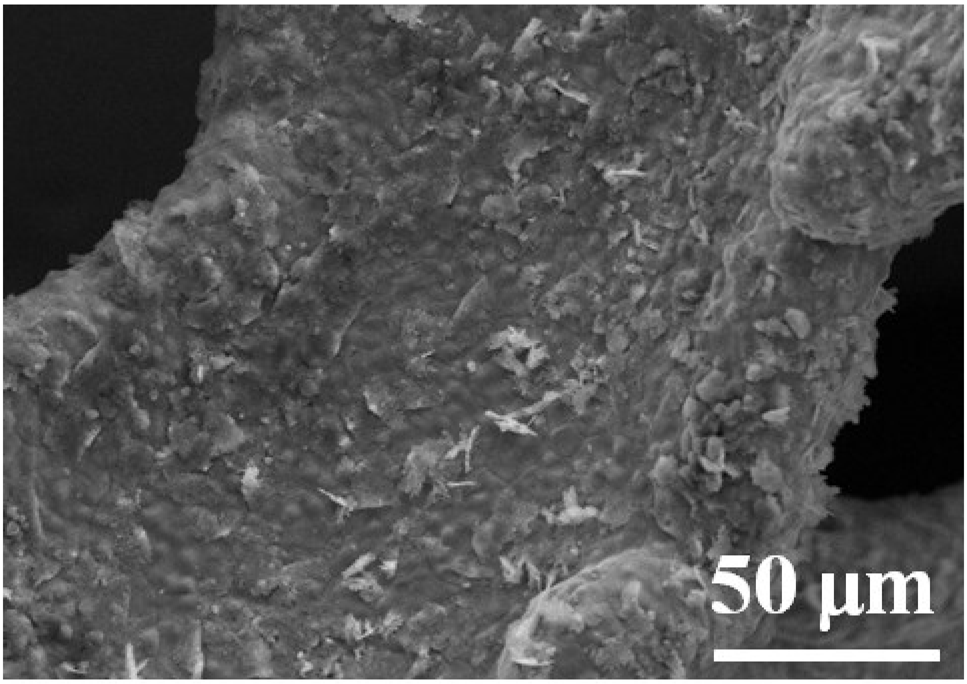


**Figure S1.** SEM image of cobalt-doped@$MnO_2$ nanosheets well distributed on nickel foam.

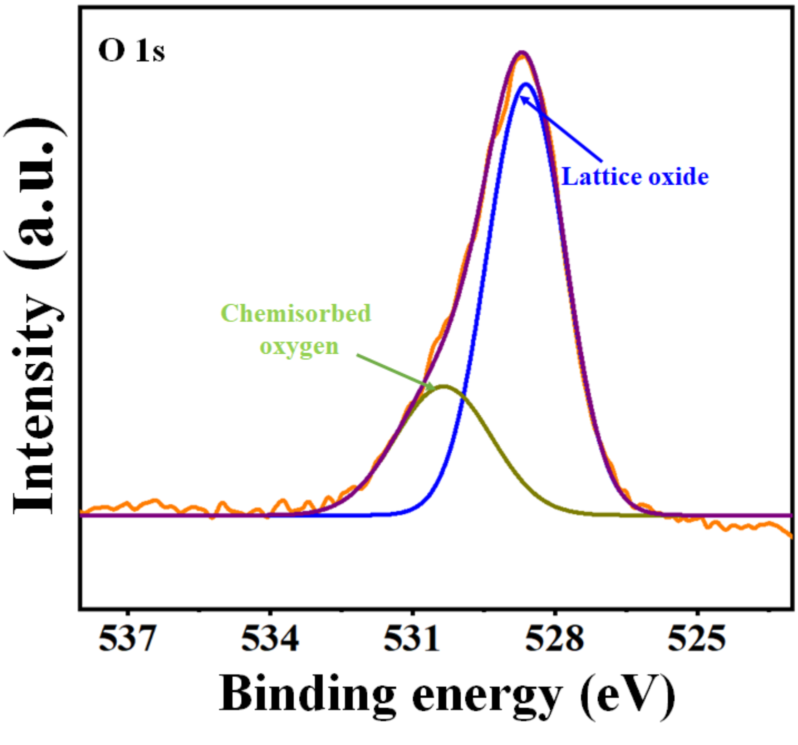


**Figure S2.** O 1s XPS spectrum of cobalt-doped@$MnO_2$ nanosheets composite.

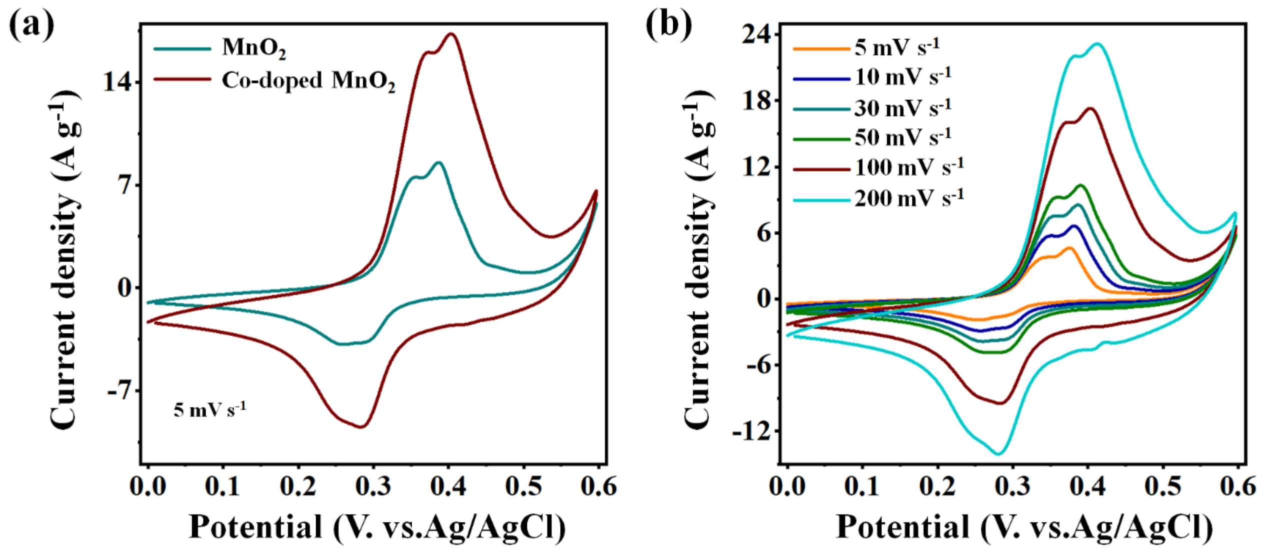


**Figure S3.** (**a**) CV comparison of $MnO_2$ and cobalt-doped@$MnO_2$ nanosheets composite electrode materials and (**b**) CV curves of $MnO_2$ sample.
